# 2-(2,4-Diphenyl-3-aza­bicyclo­[3.3.1]nonan-9-ylidenehydrazono)-1,3-thia­zolidin-4-one

**DOI:** 10.1107/S1600536809005339

**Published:** 2009-02-25

**Authors:** R. Ramachandran, M. Rani, S. Kabilan

**Affiliations:** a Department of Chemistry, Annamalai University, Annamalai Nagar 608 002, Tamil Nadu, India

## Abstract

In the title compound, C_23_H_24_N_4_OS, the piperidine and cyclo­hexane rings adopt twin chair conformations and the phenyl groups occupy equatorial positions. The dihedral angle between the two benzene rings is 10.25 (12)°. The crystal structure is stabilized by intermolecular N—H⋯O hydrogen bonds with the formation of centrosymmetric dimers.

## Related literature

For background on the thia­zolidinone system, see: Laurent *et al.* (2004 ▶). For the biological activities of thia­zolidinones, see: Shih & Ke (2004 ▶), For bicyclic compounds, see: Jeyaraman & Avila, (1981 ▶). For ring conformational analysis, see: Cremer & Pople, (1975 ▶).
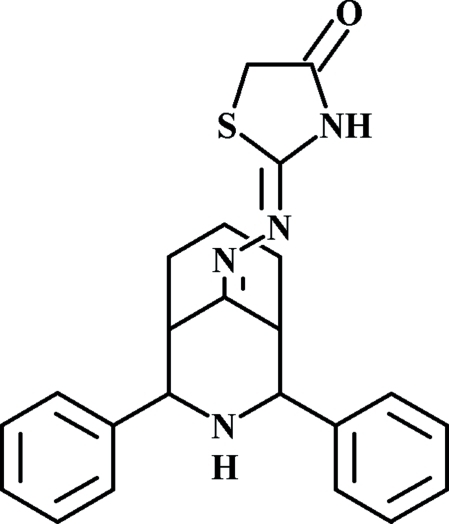


## Experimental

### 

#### Crystal data

C_23_H_24_N_4_OS
*M* *_r_* = 404.53
Monoclinic, 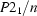

*a* = 8.3183 (3) Å
*b* = 10.8435 (4) Å
*c* = 22.7417 (8) Å
β = 92.483 (2)°
*V* = 2049.36 (13) Å^3^
*Z* = 4
Mo *K*α radiation
μ = 0.18 mm^−1^
*T* = 293 K
0.25 × 0.20 × 0.15 mm

#### Data collection

Bruker APEXII CCD diffractometer
Absorption correction: multi-scan (*SADABS*; Bruker 1999 ▶) *T*_min_ = 0.956, *T*_max_ = 0.974
31153 measured reflections
7820 independent reflections
4068 reflections with *I* > 2σ(*I*)
*R*_int_ = 0.053

#### Refinement

*R*[*F* ^2^ > 2σ(*F* ^2^)] = 0.062
*wR*(*F* ^2^) = 0.203
*S* = 1.04
7820 reflections
270 parameters
H atoms treated by a mixture of independent and constrained refinement
Δρ_max_ = 0.42 e Å^−3^
Δρ_min_ = −0.33 e Å^−3^

### 

Data collection: *APEX2* (Bruker, 2004 ▶); cell refinement: *SAINT* (Bruker, 2004 ▶); data reduction: *SAINT*; program(s) used to solve structure: *SIR92* (Altomare *et al.*, 1993 ▶); program(s) used to refine structure: *SHELXL97* (Sheldrick, 2008 ▶); molecular graphics: *ORTEP-3* (Farrugia, 1997 ▶) and *Mercury* (Macrae *et al.*, 2006 ▶); software used to prepare material for publication: *SHELXL97*.

## Supplementary Material

Crystal structure: contains datablocks global, I. DOI: 10.1107/S1600536809005339/rk2127sup1.cif

Structure factors: contains datablocks I. DOI: 10.1107/S1600536809005339/rk2127Isup2.hkl

Additional supplementary materials:  crystallographic information; 3D view; checkCIF report

## Figures and Tables

**Table 1:** Hydrogen-bond geometry (Å, °)

*D*—H⋯*A*	*D*—H	H⋯*A*	*D*⋯*A*	*D*—H⋯*A*
N4—H4*A*⋯O1^i^	0.83 (2)	2.03 (2)	2.847 (2)	169 (2)

Symmetry code: (i) 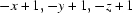.

**Fig. 1.**
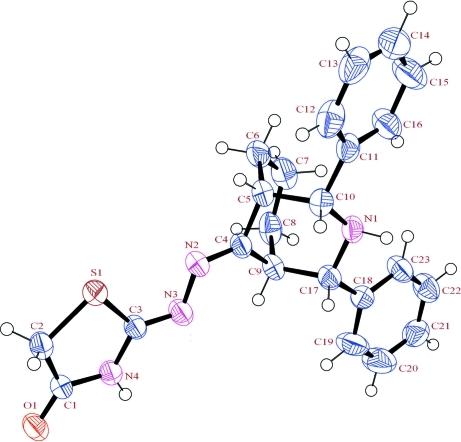
The asymmetric unit of title compound with the atom numbering scheme. Displacement ellipsoids drawn at 50% probability level. H atoms are presented as a small spheres of arbitrary radius.

**Fig. 2.**
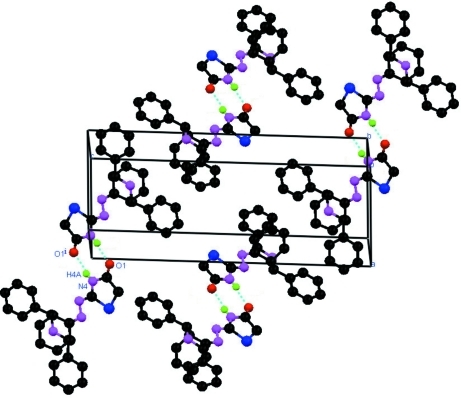
Packing of molecules in the unit cell. Hydrogen bonds are shown by dotted lines.

## supplementary crystallographic information

### Comment

Thiazolidinones are an interesting backbone unit in medicinal chemistry and is responsible for numerous pharmacological and biological properties (Shih & Ke, 2004; Laurent *et al.*, 2004), which inspires our research interest in this area towards the synthesis of thiazolidinone unit. The importance of bicyclic compounds as intermediates in the synthesis of a several physiologically active compounds have reviewed by Jeyaraman & Avila, (1981). Moreover, these bridged bicyclic compounds exhibit twin chair, chair–boat or twin boat conformations to be elucidated possessing interesting stereochemistry. In order to investigate the change in molecular conformation of piperidine and cyclohexane ring, the present investigation was made and confirmed by X–ray diffraction study.

We found, that six–membered heterocyclic piperidine ring (Fig. 1) adopt normal chair conformation with the puckering parameters (Cremer & Pople, 1975) being q_1_ and q_2_ are 0.0714 (19) Å and -0.567 (19) Å, respectively. The total puckering amplitude, Q_T_=0.572 (19) Å; θ=173.03 (19)°. Similarly, the cyclohexane ring is also adopt normal chair conformation with the puckering parameters being q_1_ and q_2_ are 0.121 (2)Å and 0.552 (2) Å, respectively. The puckering amplitude, Q_T_=0.562 (2) Å, θ=12.5 (2)°. The planar phenyl rings occupy equatorial orientation of the piperidine ring and its subtending angle between the phenyl ring and the best plane of the piperidine ring is 10.25 (12)°. The crystal structure is stabilized by intermolecular N4—H4A···O1^i^ hydrogen bonds (Fig. 2) with formation of centrosymmetrical dimers. Symmetry code: (i) -x+1, -y+1, -z+1.

### Experimental

To the boiling solution of the bicyclic thiosemicarbazone (0.01 mol) in ethanolic–chloroform (1:1 / v:v), ethylbromoacetate (0.01 mol), sodium acetate trihydrate (0.02 mol) and acetic acid few drops were added and refluxed for about 5–6 h. After the completion of reaction, excess of solvent was removed under reduced pressure and poured into water. After usual work–up, the solid was separated and purified by column chromatography using benzene–ethyl acetate (9:1 / v:v) as eluent on neutral alumina. Colourless crystals were grown by slow evaporation method using ethanol as solvent. ^1^H NMR (δ p.p.m.), DMSO–d_6_: 4.39 (s, 1H, H2a); 4.26 (s, 1H, H4a); 3.56 (s, 1H, H5e); 2.57 (s, 1H, H1e); 3.74 (s, 2H, S—CH_2_); 2.82 (m, 1H, H7a); 1.44 (m, 5H, H6e, H8e, H7e, H6a and H8a); 2.09 (s, 1H, NH at 3); 11.60 (bs, 1H, NH exchangeable); 7.25–7.60 & 7.80 (m, 10H aryl protons): ^13^C NMR (δ p.p.m.) DMSO–d_6_: 64.94 (C2); 63.57 (C4); 45.91 (C1); 39.88 (C5); 28.65 (C8); 27.28 (C6); 21.37 (C7); 32.92 (S—CH_2_); 173.98 (Cδb O); 163.00 (Cδb N) 142.54 (C2' & C4'); 128.16, 127.02, 126.95, 126.83, 126.77 (other aryl carbons).

### Refinement

The H–atoms were bonded with C atoms were placed in calculated positions and were refined using a riding model, with aromatic C—H = 0.93 Å, methine C—H = 0.98 Å, methylene C—H = 0.97 Å. The displacement parameters were set for these H atoms as *U*_iso_(H) = 1.2*U*_eq_(C). The other H atoms were found from difference Fourier map and were refined isopropically.

### Crystal data

**Table:**

C_23_H_24_N_4_OS	*F*(000) = 856
*M**_r_* = 404.53	*D*_x_ = 1.311 Mg m^−^^3^
Monoclinic, *P*2_1_/*n*	Mo *K*α radiation, λ = 0.71073 Å
Hall symbol: -P 2yn	Cell parameters from 4403 reflections
*a* = 8.3183 (3) Å	θ = 3.9–24.7°
*b* = 10.8435 (4) Å	µ = 0.18 mm^−^^1^
*c* = 22.7417 (8) Å	*T* = 293 K
β = 92.483 (2)°	Block, colourless
*V* = 2049.36 (13) Å^3^	0.25 × 0.20 × 0.15 mm
*Z* = 4	

### Data collection

**Table:**

Bruker APEXII CCD diffractometer	7820 independent reflections
Radiation source: Fine-focus sealed tube	4068 reflections with *I* > 2σ(*I*)
Graphite	*R*_int_ = 0.053
φ and ω scans	θ_max_ = 33.2°, θ_min_ = 1.8°
Absorption correction: multi-scan (*SADABS*; Bruker 1999)	*h* = −12→12
*T*_min_ = 0.956, *T*_max_ = 0.974	*k* = −16→16
31153 measured reflections	*l* = −34→34

### Refinement

**Table:**

Refinement on *F*^2^	Primary atom site location: Direct
Least-squares matrix: Full	Secondary atom site location: Difmap
*R*[*F*^2^ > 2σ(*F*^2^)] = 0.062	Hydrogen site location: Geom
*wR*(*F*^2^) = 0.203	H atoms treated by a mixture of independent and constrained refinement
*S* = 1.03	*w* = 1/[σ^2^(*F*_o_^2^) + (0.1023*P*)^2^] where *P* = (*F*_o_^2^ + 2*F*_c_^2^)/3
7820 reflections	(Δ/σ)_max_ = 0.001
270 parameters	Δρ_max_ = 0.42 e Å^−^^3^
0 restraints	Δρ_min_ = −0.33 e Å^−^^3^

### Special details

**Table:**

Geometry. All s.u.'s (except the s.u. in the dihedral angle between two l.s. planes) are estimated using the full covariance matrix. The cell s.u.'s are taken into account individually in the estimation of s.u.'s in distances, angles and torsion angles; correlations between s.u.'s in cell parameters are only used when they are defined by crystal symmetry. An approximate (isotropic) treatment of cell s.u.'s is used for estimating s.u.'s involving l.s. planes.
Refinement. Refinement of *F*^2^ against ALL reflections. The weighted *R*–factor *wR* and goodness of fit *S* are based on *F*^2^, conventional *R*–factors *R* are based on *F*, with *F* set to zero for negative *F*^2^. The threshold expression of *F*^2^ > \ s(*F*^2^) is used only for calculating *R*–factors(gt) *etc*. and is not relevant to the choice of reflections for refinement. *R*–factors based on *F*^2^ are statistically about twice as large as those based on *F*, and *R*–factors based on ALL data will be even larger.

### Fractional atomic coordinates and isotropic or equivalent isotropic displacement parameters (Å^2^)

**Table:**

	*x*	*y*	*z*	*U*_iso_*/*U*_eq_	
C1	0.6862 (2)	0.50259 (17)	0.44666 (8)	0.0387 (4)	
C2	0.8215 (2)	0.52229 (19)	0.40590 (9)	0.0463 (5)	
H2A	0.7801	0.5554	0.3686	0.056*	
H2B	0.8749	0.4447	0.3985	0.056*	
C3	0.8430 (2)	0.64074 (17)	0.50239 (8)	0.0377 (4)	
C4	1.0586 (2)	0.83875 (17)	0.58582 (8)	0.0400 (4)	
C5	1.2108 (2)	0.91183 (18)	0.58360 (9)	0.0439 (4)	
H5	1.2548	0.9015	0.5446	0.053*	
C6	1.3309 (3)	0.8579 (2)	0.62995 (11)	0.0559 (6)	
H6A	1.3594	0.7749	0.6182	0.067*	
H6B	1.4283	0.9072	0.6310	0.067*	
C7	1.2677 (3)	0.8535 (2)	0.69020 (11)	0.0627 (7)	
H7A	1.2672	0.9363	0.7063	0.075*	
H7B	1.3395	0.8038	0.7152	0.075*	
C8	1.0981 (3)	0.8002 (2)	0.69143 (10)	0.0556 (5)	
H8A	1.0554	0.8166	0.7297	0.067*	
H8B	1.1041	0.7114	0.6867	0.067*	
C9	0.9813 (2)	0.85244 (18)	0.64362 (8)	0.0430 (4)	
H9	0.8818	0.8039	0.6428	0.052*	
C10	1.1700 (2)	1.04873 (17)	0.59233 (8)	0.0421 (4)	
H10	1.1015	1.0760	0.5587	0.050*	
C11	1.3226 (2)	1.12617 (17)	0.59453 (9)	0.0442 (4)	
C12	1.4047 (3)	1.1425 (2)	0.54321 (11)	0.0620 (6)	
H12	1.3648	1.1083	0.5080	0.074*	
C13	1.5469 (3)	1.2102 (2)	0.54460 (14)	0.0733 (8)	
H13	1.6015	1.2207	0.5101	0.088*	
C14	1.6069 (3)	1.2609 (3)	0.59510 (15)	0.0796 (9)	
H14	1.7020	1.3061	0.5954	0.095*	
C15	1.5281 (3)	1.2454 (3)	0.64502 (14)	0.0804 (8)	
H15	1.5691	1.2807	0.6798	0.096*	
C16	1.3867 (3)	1.1777 (2)	0.64548 (11)	0.0612 (6)	
H16	1.3349	1.1671	0.6806	0.073*	
C17	0.9389 (2)	0.98974 (18)	0.65116 (8)	0.0420 (4)	
H17	0.8608	1.0127	0.6196	0.050*	
C18	0.8625 (2)	1.01140 (19)	0.70932 (9)	0.0433 (4)	
C19	0.7131 (3)	0.9629 (3)	0.71957 (12)	0.0766 (9)	
H19	0.6586	0.9191	0.6897	0.092*	
C20	0.6425 (3)	0.9775 (3)	0.77257 (13)	0.0835 (9)	
H20	0.5424	0.9423	0.7783	0.100*	
C21	0.7169 (3)	1.0423 (2)	0.81652 (11)	0.0594 (6)	
H21	0.6675	1.0544	0.8520	0.071*	
C22	0.8654 (3)	1.0897 (2)	0.80792 (10)	0.0584 (6)	
H22	0.9184	1.1337	0.8381	0.070*	
C23	0.9384 (3)	1.0737 (2)	0.75539 (9)	0.0534 (5)	
H23	1.0410	1.1055	0.7509	0.064*	
N1	1.08146 (19)	1.06644 (15)	0.64602 (7)	0.0421 (4)	
N2	1.01593 (19)	0.77332 (15)	0.54109 (7)	0.0433 (4)	
N3	0.87175 (19)	0.70686 (16)	0.54817 (7)	0.0456 (4)	
N4	0.70888 (19)	0.56801 (14)	0.49729 (7)	0.0403 (4)	
O1	0.57154 (16)	0.43634 (14)	0.43526 (6)	0.0525 (4)	
S1	0.96100 (5)	0.62925 (5)	0.44083 (2)	0.04262 (15)	
H1A	1.049 (3)	1.149 (2)	0.6472 (10)	0.060 (7)*	
H4A	0.635 (3)	0.569 (2)	0.5206 (11)	0.062 (7)*	

### Atomic displacement parameters (Å^2^)

**Table:**

	*U*^11^	*U*^22^	*U*^33^	*U*^12^	*U*^13^	*U*^23^
C1	0.0368 (8)	0.0358 (9)	0.0432 (10)	−0.0020 (7)	−0.0019 (7)	−0.0086 (8)
C2	0.0459 (10)	0.0462 (11)	0.0470 (11)	−0.0060 (9)	0.0046 (8)	−0.0151 (9)
C3	0.0384 (8)	0.0344 (9)	0.0400 (10)	−0.0012 (7)	−0.0040 (7)	−0.0051 (7)
C4	0.0427 (9)	0.0348 (9)	0.0418 (10)	−0.0007 (7)	−0.0065 (8)	−0.0070 (8)
C5	0.0460 (10)	0.0370 (10)	0.0485 (11)	−0.0033 (8)	−0.0007 (8)	−0.0112 (8)
C6	0.0490 (11)	0.0369 (11)	0.0804 (16)	0.0033 (9)	−0.0145 (11)	−0.0086 (10)
C7	0.0733 (15)	0.0435 (12)	0.0682 (15)	0.0093 (11)	−0.0331 (13)	−0.0022 (10)
C8	0.0817 (15)	0.0359 (11)	0.0484 (12)	0.0033 (10)	−0.0067 (11)	−0.0013 (9)
C9	0.0486 (10)	0.0375 (10)	0.0425 (10)	−0.0076 (8)	−0.0023 (8)	−0.0073 (8)
C10	0.0488 (10)	0.0380 (10)	0.0392 (10)	0.0028 (8)	0.0005 (8)	−0.0013 (8)
C11	0.0522 (10)	0.0300 (9)	0.0508 (11)	0.0024 (8)	0.0074 (9)	0.0009 (8)
C12	0.0833 (16)	0.0441 (12)	0.0604 (14)	0.0053 (11)	0.0242 (12)	0.0023 (10)
C13	0.0814 (17)	0.0487 (14)	0.093 (2)	0.0055 (13)	0.0471 (16)	0.0057 (14)
C14	0.0651 (15)	0.0590 (16)	0.117 (3)	−0.0164 (13)	0.0349 (16)	−0.0108 (16)
C15	0.0707 (15)	0.0826 (19)	0.089 (2)	−0.0358 (14)	0.0130 (14)	−0.0197 (16)
C16	0.0637 (13)	0.0619 (14)	0.0589 (14)	−0.0231 (11)	0.0139 (11)	−0.0126 (12)
C17	0.0425 (9)	0.0408 (10)	0.0420 (10)	−0.0008 (8)	−0.0049 (8)	−0.0053 (8)
C18	0.0391 (9)	0.0439 (11)	0.0464 (11)	0.0021 (8)	−0.0045 (8)	−0.0072 (8)
C19	0.0392 (11)	0.120 (2)	0.0705 (16)	−0.0151 (13)	0.0006 (11)	−0.0420 (16)
C20	0.0465 (12)	0.121 (3)	0.0839 (19)	−0.0169 (15)	0.0155 (12)	−0.0345 (18)
C21	0.0557 (12)	0.0656 (15)	0.0574 (13)	0.0076 (11)	0.0095 (10)	−0.0089 (12)
C22	0.0664 (14)	0.0641 (14)	0.0443 (12)	−0.0100 (11)	−0.0024 (10)	−0.0130 (10)
C23	0.0533 (11)	0.0563 (13)	0.0504 (12)	−0.0155 (10)	0.0000 (10)	−0.0109 (10)
N1	0.0483 (8)	0.0304 (8)	0.0479 (9)	−0.0011 (7)	0.0056 (7)	−0.0043 (7)
N2	0.0424 (8)	0.0402 (9)	0.0468 (9)	−0.0052 (7)	−0.0035 (7)	−0.0089 (7)
N3	0.0466 (8)	0.0466 (9)	0.0435 (9)	−0.0090 (7)	0.0003 (7)	−0.0097 (7)
N4	0.0378 (8)	0.0414 (9)	0.0421 (9)	−0.0047 (7)	0.0053 (7)	−0.0088 (7)
O1	0.0470 (7)	0.0553 (9)	0.0553 (9)	−0.0150 (6)	0.0028 (6)	−0.0171 (7)
S1	0.0391 (2)	0.0403 (3)	0.0487 (3)	−0.00407 (19)	0.00437 (19)	−0.0065 (2)

### Geometric parameters (Å, °)

**Table:**

C1—O1	1.213 (2)	C11—C16	1.373 (3)
C1—N4	1.359 (2)	C11—C12	1.389 (3)
C1—C2	1.504 (3)	C12—C13	1.391 (4)
C2—S1	1.8009 (19)	C12—H12	0.9300
C2—H2A	0.9700	C13—C14	1.349 (4)
C2—H2B	0.9700	C13—H13	0.9300
C3—N3	1.278 (2)	C14—C15	1.346 (4)
C3—N4	1.367 (2)	C14—H14	0.9300
C3—S1	1.7487 (18)	C15—C16	1.387 (3)
C4—N2	1.278 (2)	C15—H15	0.9300
C4—C9	1.496 (3)	C16—H16	0.9300
C4—C5	1.496 (3)	C17—N1	1.458 (2)
C5—C6	1.537 (3)	C17—C18	1.510 (3)
C5—C10	1.538 (3)	C17—H17	0.9800
C5—H5	0.9800	C18—C23	1.377 (3)
C6—C7	1.489 (3)	C18—C19	1.379 (3)
C6—H6A	0.9700	C19—C20	1.372 (3)
C6—H6B	0.9700	C19—H19	0.9300
C7—C8	1.526 (3)	C20—C21	1.350 (4)
C7—H7A	0.9700	C20—H20	0.9300
C7—H7B	0.9700	C21—C22	1.360 (3)
C8—C9	1.535 (3)	C21—H21	0.9300
C8—H8A	0.9700	C22—C23	1.375 (3)
C8—H8B	0.9700	C22—H22	0.9300
C9—C17	1.541 (3)	C23—H23	0.9300
C9—H9	0.9800	N1—H1A	0.94 (3)
C10—N1	1.466 (2)	N2—N3	1.414 (2)
C10—C11	1.521 (3)	N4—H4A	0.83 (2)
C10—H10	0.9800		
			
O1—C1—N4	124.69 (17)	C16—C11—C12	118.0 (2)
O1—C1—C2	123.70 (17)	C16—C11—C10	123.06 (18)
N4—C1—C2	111.60 (15)	C12—C11—C10	118.9 (2)
C1—C2—S1	107.74 (13)	C11—C12—C13	119.8 (3)
C1—C2—H2A	110.2	C11—C12—H12	120.1
S1—C2—H2A	110.2	C13—C12—H12	120.1
C1—C2—H2B	110.2	C14—C13—C12	121.2 (2)
S1—C2—H2B	110.2	C14—C13—H13	119.4
H2A—C2—H2B	108.5	C12—C13—H13	119.4
N3—C3—N4	121.05 (17)	C15—C14—C13	119.4 (2)
N3—C3—S1	126.91 (14)	C15—C14—H14	120.3
N4—C3—S1	112.04 (13)	C13—C14—H14	120.3
N2—C4—C9	129.74 (17)	C14—C15—C16	121.1 (3)
N2—C4—C5	118.26 (17)	C14—C15—H15	119.4
C9—C4—C5	111.96 (15)	C16—C15—H15	119.4
C4—C5—C6	107.51 (17)	C11—C16—C15	120.5 (2)
C4—C5—C10	108.35 (16)	C11—C16—H16	119.8
C6—C5—C10	114.83 (16)	C15—C16—H16	119.8
C4—C5—H5	108.7	N1—C17—C18	110.86 (15)
C6—C5—H5	108.7	N1—C17—C9	110.57 (15)
C10—C5—H5	108.7	C18—C17—C9	110.78 (16)
C7—C6—C5	113.48 (18)	N1—C17—H17	108.2
C7—C6—H6A	108.9	C18—C17—H17	108.2
C5—C6—H6A	108.9	C9—C17—H17	108.2
C7—C6—H6B	108.9	C23—C18—C19	116.5 (2)
C5—C6—H6B	108.9	C23—C18—C17	123.12 (18)
H6A—C6—H6B	107.7	C19—C18—C17	120.33 (18)
C6—C7—C8	113.11 (19)	C20—C19—C18	121.8 (2)
C6—C7—H7A	109.0	C20—C19—H19	119.1
C8—C7—H7A	109.0	C18—C19—H19	119.1
C6—C7—H7B	109.0	C21—C20—C19	120.7 (2)
C8—C7—H7B	109.0	C21—C20—H20	119.6
H7A—C7—H7B	107.8	C19—C20—H20	119.6
C7—C8—C9	113.88 (18)	C20—C21—C22	118.7 (2)
C7—C8—H8A	108.8	C20—C21—H21	120.7
C9—C8—H8A	108.8	C22—C21—H21	120.7
C7—C8—H8B	108.8	C21—C22—C23	121.1 (2)
C9—C8—H8B	108.8	C21—C22—H22	119.5
H8A—C8—H8B	107.7	C23—C22—H22	119.5
C4—C9—C8	107.61 (17)	C22—C23—C18	121.2 (2)
C4—C9—C17	107.63 (16)	C22—C23—H23	119.4
C8—C9—C17	114.75 (16)	C18—C23—H23	119.4
C4—C9—H9	108.9	C17—N1—C10	115.60 (15)
C8—C9—H9	108.9	C17—N1—H1A	108.1 (14)
C17—C9—H9	108.9	C10—N1—H1A	107.7 (14)
N1—C10—C11	110.42 (15)	C4—N2—N3	113.58 (17)
N1—C10—C5	110.85 (16)	C3—N3—N2	108.82 (16)
C11—C10—C5	110.40 (16)	C1—N4—C3	117.12 (16)
N1—C10—H10	108.4	C1—N4—H4A	118.2 (17)
C11—C10—H10	108.4	C3—N4—H4A	124.0 (17)
C5—C10—H10	108.4	C3—S1—C2	91.47 (8)
			
O1—C1—C2—S1	178.98 (16)	C4—C9—C17—N1	−55.7 (2)
N4—C1—C2—S1	−1.0 (2)	C8—C9—C17—N1	64.0 (2)
N2—C4—C5—C6	−114.0 (2)	C4—C9—C17—C18	−179.00 (15)
C9—C4—C5—C6	64.1 (2)	C8—C9—C17—C18	−59.3 (2)
N2—C4—C5—C10	121.3 (2)	N1—C17—C18—C23	−13.3 (3)
C9—C4—C5—C10	−60.5 (2)	C9—C17—C18—C23	109.8 (2)
C4—C5—C6—C7	−55.1 (2)	N1—C17—C18—C19	169.9 (2)
C10—C5—C6—C7	65.6 (2)	C9—C17—C18—C19	−66.9 (3)
C5—C6—C7—C8	47.2 (2)	C23—C18—C19—C20	1.0 (4)
C6—C7—C8—C9	−46.0 (3)	C17—C18—C19—C20	177.9 (3)
N2—C4—C9—C8	115.1 (2)	C18—C19—C20—C21	1.2 (5)
C5—C4—C9—C8	−62.8 (2)	C19—C20—C21—C22	−2.0 (5)
N2—C4—C9—C17	−120.7 (2)	C20—C21—C22—C23	0.8 (4)
C5—C4—C9—C17	61.4 (2)	C21—C22—C23—C18	1.4 (4)
C7—C8—C9—C4	52.2 (2)	C19—C18—C23—C22	−2.2 (3)
C7—C8—C9—C17	−67.6 (2)	C17—C18—C23—C22	−179.0 (2)
C4—C5—C10—N1	53.5 (2)	C18—C17—N1—C10	177.21 (15)
C6—C5—C10—N1	−66.7 (2)	C9—C17—N1—C10	54.0 (2)
C4—C5—C10—C11	176.21 (16)	C11—C10—N1—C17	−175.50 (16)
C6—C5—C10—C11	56.0 (2)	C5—C10—N1—C17	−52.8 (2)
N1—C10—C11—C16	15.8 (3)	C9—C4—N2—N3	1.4 (3)
C5—C10—C11—C16	−107.2 (2)	C5—C4—N2—N3	179.20 (16)
N1—C10—C11—C12	−166.51 (18)	N4—C3—N3—N2	−179.13 (16)
C5—C10—C11—C12	70.6 (2)	S1—C3—N3—N2	1.1 (2)
C16—C11—C12—C13	−0.4 (3)	C4—N2—N3—C3	−177.32 (17)
C10—C11—C12—C13	−178.2 (2)	O1—C1—N4—C3	−178.36 (18)
C11—C12—C13—C14	−0.1 (4)	C2—C1—N4—C3	1.6 (2)
C12—C13—C14—C15	0.2 (4)	N3—C3—N4—C1	178.78 (18)
C13—C14—C15—C16	0.3 (5)	S1—C3—N4—C1	−1.5 (2)
C12—C11—C16—C15	0.9 (4)	N3—C3—S1—C2	−179.58 (19)
C10—C11—C16—C15	178.7 (2)	N4—C3—S1—C2	0.67 (15)
C14—C15—C16—C11	−0.9 (5)	C1—C2—S1—C3	0.16 (15)

### Hydrogen-bond geometry (Å, °)

**Table:**

*D*—H···*A*	*D*—H	H···*A*	*D*···*A*	*D*—H···*A*
N4—H4A···O1^i^	0.83 (2)	2.03 (2)	2.847 (2)	169 (2)

Symmetry codes: (i) −*x*+1, −*y*+1, −*z*+1.
